# Daily Challenges and Resources of Adults with Chronic Dysphagia: A Qualitative Investigation

**DOI:** 10.1007/s00455-024-10764-5

**Published:** 2024-10-08

**Authors:** Aurora Ninfa, Giulia Morandi, Antonio Schindler, Antonella Delle Fave

**Affiliations:** 1 Department of Pathophysiology and Transplantation, University of Milan, Milan, Italy; 2 Department of Biomedical Sciences, University of Milan, Milan, Italy

**Keywords:** Swallowing disorders, Dysphagia, Chronicity, Positive adjustment, Daily experience

## Abstract

Identifying and addressing daily challenges and resources associated with chronic oropharyngeal dysphagia (OD) is a pivotal, though still neglected component of person-centred care, yet overlooked in research studies. To investigate these dimensions, 25 Italian adults with chronic OD due to cancer or neurodegenerative diseases participated in semi-structured interviews, designed following a modified framework analysis approach. Two researchers independently transcribed and coded interviews, elaborated a working analytical framework, indexed and charted the data, solving discrepancies through negotiated agreement and discussion with a third researcher. Proportion agreement on extracted quotations was calculated. Overall, 457 quotations were extracted from the interviews (88% agreement). Daily challenges pertained to physical, practical, and social domains; most participants reported OD-related problems; almost half mentioned care needs and obstacles in using healthcare services. Concerning resources in OD management, most participants referred to problem-focused and meaning-focused coping strategies, personal capabilities, and support from family and healthcare services. Finally, almost half of the participants reported OD-related changes in life view and meaning. Findings suggest that adjusting to OD implies challenges and resource mobilization in different life domains. Future studies should longitudinally elucidate the dynamics of positive adjustment, to promote patient-centred OD care based on individually perceived needs and challenges, and to inform healthcare policies.

## Introduction

Oropharyngeal dysphagia (OD) consists of any alteration of the oral or pharyngeal phase of the swallowing physiology [1], that may occur as a consequence of different health conditions [2]. Unsolvable and progressive sensory-motor alterations of the swallowing system related to head and neck cancers (HNC) treated with (chemo)radiotherapy or neurodegenerative conditions may lead to chronic OD [3–6], namely oropharyngeal swallowing disorders which persist for more than 6 months and due to their aetiology are not likely to resolve in time. Possible clinical consequences of OD include aspiration pneumonia [7], malnutrition, and dehydration [8, 9]. At the psychosocial level, people with OD may experience limitations in daily meal consumption due to the swallowing problem or the dietary modifications prescribed for its management [10, 11]. These limitations include having reduced physical safety, reduced choice and control, and poor mealtime experiences [12]. Due to these limitations, people with OD may avoid social gatherings which involve consuming food and beverages and may struggle with feelings of loss, anxiety, and depression [13]. Two systematic reviews showed that these negative consequences reduce individuals’ health-related quality of life (HRQOL) proportionally to symptoms’ severity and intrusiveness of dietary modifications [14, 15].

**Fig. 1:**
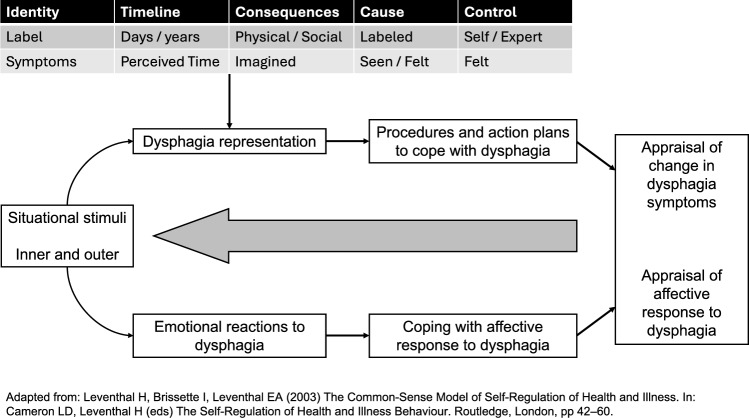
The common-sense model of self-regulation adapted to people with dysphagia. Adapted from: Leventhal H, Brissette I, Leventhal EA (2003) The Common-Sense Model of Self-Regulation of Health and Illness. In: Cameron LD, Leventhal H (eds) The Self-Regulation of Health and Illness Behaviour. Routledge, London, pp 42–60

The mere focus on challenges and limitations is however insufficient to comprehensively capture the person’s experience of disease. The International Classification of Functioning, Disability, and Health (ICF) [16–18] promoted a broadened vision from disease to person and from impairments to resources. According to the ICF, individuals’ health condition results from the interplay among impairments of body functions and structures (in the case of OD, the swallowing function), daily activities (eating and drinking), and social participation (social eating). Environmental factors (e.g., (caregiver’s support, utensils, modified-texture diets) and personal factors (e.g., personality, coping strategies, cultural norms and values) are included as potential barriers and facilitators of individual functioning and health. In addition, research in Positive Psychology provided evidence of the resources and constructive dimensions of human experience, identifying personal and social resources associated with positive adjustment to disease [19, 20]. Personal resources include coping strategies [21], resilient behaviours [22], hardiness [23], self-efficacy [24], posttraumatic growth [25], the cultivation of hope [26], and optimism [27]. Among social resources, the support received by informal caregivers and healthcare services plays a pivotal role [28]. The care needs and resources perceived by people with OD have been rarely explored. Moreover, the adoption of theoretically sound, patient-centred frameworks can help interpret related results from the perspective of patients’ experiences. Particularly relevant to this aim is the Supportive Care Framework (SCF) [29], developed to classify the individual needs of patients diagnosed with cancer in order to provide appropriate and timely services. The SCF was adapted to individuals with OD and their informal caregivers, and their care needs were classified into five domains: physical, practical, informational, psychological, and social [13]. Another valuable model to understand and contextualize the daily challenges and resources perceived by patients with chronic OD is the Common-Sense Model of Self-Regulation (CSM) [30, 31]. The core assumption of CSM adapted to people with OD is that individuals develop personal beliefs about their disorder (dysphagia representations), its management (treatment representations), and the related affective response (emotional reactions to dysphagia). Based on these beliefs and representations, individuals identify and implement action plans to cope with OD. The dysphagia and treatment representations are articulated into five components: label and symptoms (identity); causes; course and duration (i.e., acute, chronic, or cyclic timeline); expected effects on the individual’s life (consequences); availability of interventions to affect dysphagia course through personal actions (controllability) and medical treatments (curability). Figure 1 shows the components of the CSM adapted to people with OD. Coping responses to OD may be categorized according to the Stress and Coping theory [21] as problem-focused (i.e., aimed at solving the perceived OD symptoms, and limitations), emotion-focused (i.e., aimed at dealing with the emotions associated with OD), meaning-focused (i.e., aimed at attributing a meaning to OD and its consequences), and avoidance coping (i.e., aimed at avoiding OD and its consequences). Noticeably, this self-regulation system is shaped as a meaningful, coherent, and comprehensible individual narrative. Thus, patients’ representations of and expectations about dysphagia and its treatment may not be congruent with clinicians’ evaluations.

These models and related findings suggest the importance of addressing the challenges and supporting the resources perceived by persons with chronic OD, a rather overlooked topic in research, intervention and healthcare policies. The first step to attain this goal is to understand how people with chronic OD experience daily challenges and mobilise personal and social resources, through the lens of sound theoretical frameworks. The interplay between well-established theories in the field of health psychology and individual narratives of people with chronic OD could provide useful information to design and develop person-centred OD care.

This study was therefore aimed to contribute filling a gap in the literature, by investigating daily challenges and resources perceived by adults living with chronic OD.

Based on a recent scoping review and the ICF framework [13, 32], we hypothesized that participants would report challenges across a variety of domains beyond the physical one, including practical, social, psychological, and informational issues, as well as a broad set of social and personal resources.

## Methods

To understand the nature and relevance of OD-related challenges and resources through participants’ direct experiences in real-life settings [33], a qualitative observational, cross-sectional study was conducted, adopting a pragmatist perspective to design procedures and interpret findings. A modified framework analysis was chosen to combine the deductive analytic approach of framework analysis with aspects from a Grounded Theory perspective, which included open coding, axial coding, and constant comparison [34, 35]. Through this pragmatist approach, qualitative information (inductive perspective) was framed into existing theoretical frameworks (deductive perspective) and used to expand them by further grounding the frameworks in participants’ experiences. The study protocol was approved by the Institutional Review Board of the Luigi Sacco Hospital (2020/ST/018).

### Participants

Participants were recruited among individuals with known or suspected OD attending outpatient services at a Phoniatrics clinic in Northern Italy, between November 2019 and September 2022. Inclusion criteria were (i) dysphagia at the Fiberoptic Endoscopic Evaluation of Swallowing (Dysphagia Outcome and Severity Scale ≤ 5), (ii) diagnosis of Head and Neck Cancer or neurodegenerative diseases, (iii) age ≥ 18 years. Exclusion criteria were (i) comorbidity with psychiatric conditions or cognitive decline (Mini Mental State Examination < 24), (ii) insufficient comprehension and fluency in Italian. Purposeful sampling with a maximum variation strategy [36] (to guarantee the representation of different diagnoses, ages, and OD severity) was used to select participants. Sampling ended at the saturation of the themes that emerged in the interviews (i.e., no new information could be collected through additional interviews). All participants signed informed consent.

### Measures

Semi-structured interviews were used to explore participants’ experiences of OD-related challenges and care resources in daily life. The 5-step process proposed by Kallio et al. [37] guided the development of the interview. The appropriateness of using semi-structured interviews for this study was justified by the epistemological paradigm adopted (*Step 1*). Semi-structured interviews allow to develop and expand theoretical frameworks through a bottom-up process, by rigorously exploring the phenomenon of interest through a focused discussion (collecting similar types of information across participants) [38] while enabling reciprocity between the interviewer and the participant [39]. The structure of the interview was based on existing theoretical models and empirical evidence (*Step 2*). In particular, the assumption that persons with OD may experience challenges which are not limited to the physical function but cover several life domains was supported by a recent scoping review on the topic [13]. The theory of Stress and Coping [21] and the Common Sense Model of Self-Regulation [30, 40] provided a solid background to the expectation that individuals adopt coping strategies consistent with the subjective representation of their problem (OD in this study). The assumption that problems and resources in dealing with health issues include personal and environmental factors is based on the International Classification of Functioning, Disability, and Health (ICF) [16–18]. Finally, the possibility that a chronic health condition may lead to changes in one’s life vision is grounded in the Response Shift Theory [41]. A preliminary semi-structured interview guide including 13 questions was formulated by two researchers with expertise in qualitative methods, subjective experience of chronic conditions, and oropharyngeal dysphagia (*Step 3*). Questions were clearly worded and mostly open-ended, guiding participants through a logical and coherent narration of their own experiences. After a preliminary field-testing of the interview guide (*Step 4*), four questions eliciting redundant answers were removed, the syntactic and semantic structure of three questions was simplified, and the medical term “dysphagia” was replaced with the patient-centred wording “swallowing problems”. The final semi-structured interview guide, presented as supplementary material (Appendix 1), included 9 questions (*Step 5*). The following themes were covered: (1) challenges related to living with swallowing problems; (2) strategies used to address these issues; (3) sources of these strategies (personal experience and/or suggestions from others); (4) personal capabilities used to cope with swallowing problems, (5) social and family role limitations related to swallowing problems; (6) support from family or significant others; (7) support from healthcare services; (8) obstacles in accessing healthcare services and possible solutions; and (9) changes in one’s life view triggered by the swallowing problem. Each interview started with a brief description of the aims of the conversation. Complementary questions were added when necessary, inviting participants to expand on their opinions or to clarify ambiguous statements. Any additional comment or remark provided by participants prior to closing the interview was collected as well.

### Procedures

After approval of the study protocol by the local Institutional Review Board, eligible participants were identified based on the established inclusion and exclusion criteria. A Phoniatric assessment with Fiberoptic Endoscopic Evaluation of Swallowing was conducted to objectify the presence of OD (Dysphagia Outcome and Severity Scale [42] ≤ 5), and a screening of cognitive functions (Mini Mental State Examination [43] ≥ 24) was performed to detect the absence of cognitive decline. All participants signed an informed consent, which detailed the study aims and procedures, including the audio recording of the interviews, the pseudonymisation of collected data, and the right to freely withdraw from the study without any consequence for their clinical healthcare management. Semi-structured interviews were conducted by a Speech Language Pathologist (SLP) with 5 years of clinical experience and 4 years of expertise in dysphagia assessment and in qualitative investigation of health-related quality of life and well-being. Based on each participant’s preference, interviews took place in person at the outpatient clinic or through video calls with participants in a comfortable and silent place of their choice. A protected and non-judgmental environment was ensured by the interviewer’s active listening of participants’ opinions through verbal and non-verbal probing, and by reminding participants of the absence of right and wrong answers. Audio tracks of the interviews were recorded using the software GarageBand (version 10.1.0) on a MacBook Pro and exported in.mp4 format. Recordings were transcribed *verbatim* and checked for accuracy by a second SLP researcher with expertise in OD and qualitative methodology.

Participants’ demographic data (age, sex, education, working status, living arrangement, and sources of social support), clinical data (diagnosis, time from diagnosis, time from OD onset, feeding modality, use of OD-related health services), and research data (interviews) were recorded, de-identified through pseudonymisation, and stored on an institutional hard drive.

### Data Analysis

The data analysis process followed the stages of modified framework analysis [34]. Two SLP researchers transcribed and familiarized themselves with participants’ narratives (Stages 1–2); subsequently, they independently extracted meaningful text units (quotations) on a Microsoft Excel sheet (Microsoft Corporation, 2018) using an inductive open coding (Stage 3); through axial coding they refined key categories and identified relationships between them, organizing quotations into categories and sub-categories pertaining to each question theme. Using the iterative process of constant comparison, categories and subcategories were further refined based on newly collected data, and a working analytical framework was developed which linked participants’ narratives with the theoretical frameworks of Supportive Care Framework adapted to OD [13], the International Classification of Functioning Disability and Health[16], and the Common Sense Model of Self-Regulation [30, 40] (Stage 4). Then, the working analytical framework was applied by indexing transcripts with existing codes and categories (Stage 5) and data were charted (Stage 6). These stages were collaborative and iterative, the interpretation of codes and themes was shared and discussed among the two SLP researchers and, if consensus could not be reached (negotiated agreement), with a third senior academic researcher with expertise in health psychology (Stage 7). The same strategy (discussion and consultation with a third researcher in case of disagreement) was used in Stage 3 on extracted quotations. As an additional quantitative indicator, the proportion agreement between coders was calculated by computing the percentage of agreement on extracted quotations. A total of 457 meaningful text units were extracted from participants’ interviews; 404 of them were extracted by both coders. The total proportion agreement (88%) and proportion agreement for single questions (82% to 100%, detailed in Appendix 2) showed acceptable levels since they exceeded the threshold of 80% [44].

Descriptive statistics included the absolute frequency and percentage distribution of quotations pertaining to each category and subcategory, both on the total of collected answers and on the number of participants. In addition, to represent participants’ voices, exemplary quotations were included. To enhance clarity and readability while ensuring adherence to participants’ perspective, exemplary quotations were first translated from Italian to English by the researcher who conducted the interviews, and subsequently revised for language use by an experienced academic researcher and a native English speaker.

## Results

Among the 57 patients with chronic OD who met the inclusion criteria, 25 were purposefully selected for the present study. The remaining 32 eligible individuals were excluded due to previous inclusion of participants with similar diagnoses, age, or dysphagia severity, or when saturation of the interview themes occurred. Table 1 provides information on participants’ demographic and clinical features. Most participants were males in their sixties, with an average schooling of 13 years; only a minority of them were employed. The vast majority cohabited, perceived support from their family members and cultivated religious beliefs. Almost half of the participants were diagnosed with HNC and treated with chemo-radiotherapy (n = 9, 36%) or radiotherapy (n = 3, 12%). The other participants acquired OD as a consequence of neurodegenerative conditions. At the time of data collection, participants had been experiencing swallowing problems on average for 4 years; their feeding adaptation patterns included removing hard-to-swallow food from diet (n = 10, 40%), preparing the food in a soft, easy-to-chew fashion (n = 8, 32%), restricting full oral intake to one consistency (n = 3, 12%), or using a Percutaneous Endoscopic Gastrostomy (PEG) for the majority of their nutritional intake (n = 2, 8%). Only two (8%) participants did not feel the need for changes in feeding patterns. Regarding health services used to cope with OD, prior to the study 11 participants (44%) had undergone SLP sessions, in the form of either counselling or rehabilitation exercises, while 4 (16%) were continuing SLP rehabilitation at the time of data collection. In addition, 7 participants (28%) used psychological support services to cope with the disease which caused OD (n = 5, 72%), to cope with OD itself (n = 1, 14%), or to deal with other personal issues (n = 1, 14%).

**Table 1:** Participants’ demographic and clinical characteristics

N = 25	N	% or mean ± SD (min–max)
Age (years)	25	66.4 ± 14.2 (33–86)
Sex		
Male	16	64%
Female	9	36%
Education (years)	25	12.9 ± 3.9 (5–20)
Work		
Employed	7	28%
Retired	9	36%
Invalidity pension	3	12%
Unemployed	6	24%
Living arrangements		
Living together	23	92%
Living alone	2	8%
Source of social support (multiple answers)		
Family	19	76%
Friends	4	16%
Colleagues	4	16%
Other	1	4%
None	6	24%
Diagnosis		
HNC	12	48%
Neurodegenerative diseases^a^	13	52%
Time from diagnosis (months)	24	87.6 ± 60.7 (19–240)
Time from OD onset (months)	25	48.7 ± 42.8 (6–168)

*OD* oropharyngeal dysphagia. *HNC* head and neck cancer

^a^Huntington’s disease (N = 2, 8%); Oculopharyngeal dystrophy (N = 2, 8%); Steinert myotonic dystrophy (N = 2, 8%); Charcot-Marie-Tooth disease (N = 2, 8%); Kennedy’s disease (N = 1, 4%); Alexander’s disease (N = 1, 4%); finally, idiopathic OD (N = 3, 12%)

Most interviews were performed through video calls (19 participants, 76%), while the remaining 6 (24%) were conducted in person at the outpatient clinic. All participants completed the interview.

### OD-related Challenges

All participants (N = 25) answered the question about OD-related challenges (105 quotations extracted), primarily reporting problems or limitations related to OD daily management (n = 24, 96%). Only one participant (4%) did not describe any issue in coping with OD. Based on their contents, challenges were categorized as physical, practical, psychological, and social. Physical problems and limitations mostly comprised symptoms characterizing OD, difficulties in swallowing specific foods, and the unpleasant taste of safely edible food. Practical issues mostly concerned mealtime management and the dearth of food with adequate characteristics when eating out. In the psychological domain, participants emphasized in similar proportions the loss of eating enjoyment, difficulties in accepting their condition, managing specific daily situations, and coping with negative emotions. At the social level, they were mostly concerned with changes in their social role because of the reduced participation in convivial occasions.

Besides problems and limitations, 12 participants (48%) reported OD-related care needs. At the physical level, needs concerned texture-modified tasty food; at the practical level, improvements in meal management; and at the psychological level, more effective strategies to cope with negative emotions and to cultivate hope. Table 2 shows the frequency and percentage distribution of categories and subcategories related to the challenges reported by the participants, with sample quotations.

**Table 2:** Categorization of perceived OD-related challenges (question 1)

Category	Subcategory	Specific themes	n (%)	Ref (%)	Citations
Problems and limitations	Physical		19 (76%)	46 (44%)	
		Swallowing function	15 (60%)	20 (19%)	“If I talk [while eating], I may cough”
		Food characteristics	12 (48%)	13 (12%)	“Meatballs or a steak, I cannot eat them anymore. Apples […], coconuts I cannot”
		Oral cavity	4 (16%)	5 (5%)	“I have much phlegm”
		Taste	3 (12%)	4 (4%)	“Eating everything in a mush shape weighs on me”
		Dysphagia complications	2 (8%)	2 (2%)	“Only in the last few years, this problem of pneumonia came up”
		Speaking	1 (4%)	2 (2%)	“Everything gets slowed down […] the efficacy of conversations”
	Practical		11 (44%)	20 (19%)	
		Meal management	8 (32%)	13 (12%)	“Mealtimes […] last one hour, one hour and a quarter […] it is really hard. When it’s over, I’m exhausted”
		Time management	4 (16%)	5 (5%)	“I’m forced to sit down to eat, eating on the run a sandwich, with mortadella and that stuff, I cannot do it”
		Food availability	2 (8%)	2 (2%)	“[At] restaurants I cannot eat what others eat”
	Psychological		4 (16%)	6 (6%)	
		Acceptance	1 (4%)	2 (2%)	“It’s difficult to get used to it. I think [swallowing] will not be normal again”
		Emotion-focused coping	1 (4%)	1 (1%)	“You always have fear, maybe you cough, you say “Here we are” and then nothing happens”
		Problem-focused coping	1 (4%)	2 (2%)	“I am aware of what to do [to manage the swallowing problem]. Unfortunately, many times I don’t manage to do it, it’s my fault. It’s [Huntington’s] disease fault”
		Eating enjoyment	1 (4%)	1 (1%)	“You lose eating enjoyment […] all pureed stinks”
	Social		10 (40%)	15 (14%)	
		Change in social roles	8 (32%)	12 (11%)	“I cannot allow myself to talk while eating […] I say nothing, I cannot participate at all in a discussion because if I try to answer or have my say, I must interrupt chewing”
		Dyad member’s well-being	2 (8%)	3 (3%)	“I feel sorry for my family […] sometimes I feel guilty […] they cannot give up [on social occasions] because of me”
	None	-	1 (4%)	1 (1%)	“I don’t have limitations because I can eat everything”
Needs	Physical	Food characteristics	5 (20%)	6 (6%)	“[I need to] eat soft meals […] more sauce if you eat pasta, more bechamel if you eat lasagna”
	Practical		6 (24%)	9 (9%)	
		Meal management	6 (24%)	7 (7%)	“I have to think about the shopping, what to buy, how to cook and process it, and how to put it on the table”
		Time management	1 (4%)	1 (1%)	“[There is a need for] organizing your day […] because after [in the evening] I have to take the PEG”
		Swallowing team	1 (4%)	1 (1%)	“It is important to me to have a point of reference [for the management of swallowing]”
	Psychological		1 (4%)	2 (2%)	
		Emotion-focused coping	1 (4%)	1 (1%)	“I wish, Doctor, I was different to express my emotions […] the problem is [that] if I shut myself up, the situation worsens”
		Hope	1 (4%)	1 (1%)	“I wish […] [that] changing the feeding modality in that way [PEG] could help me regaining weight, physical and also psychological shape”

n = number of participants who cited the category/subcategory. Ref = number of text units retrieved for each category/subcategory

When specifically asked about social limitations related to OD (question 5) all participants referred to family and social situations (53 extracted quotations). More specifically, nine participants (36%; quotations: n = 9, 17%) reported limitations and changes in their family role *(“I miss a little my presence [in the family] […] not physically […] I mean, I miss the presence as husband, as son, as father”*) and—to a lesser extent—lack of support by family members (*“Sometimes I get angry with whom is at home with me […] because they don’t wait for me. I say to them: “[…] don’t make me always feel the last finishing the dinner”*) and worries about the spouse’s well-being (*“My husband, when […] I start [coughing], if we are eating, he stops with his eyes open wide, because sometimes he saw I was feeling bad”*). Ten (40%) participants did not feel limited in their family life in any respect (quotations n = 10, 19%), stating *“The family life does not change much”*.

As regards social life, almost half of the participants (n = 11, 44%; quotations n = 20, 38%) perceived limitations due to changes in their social role (*“It is difficult to go out even for a coffee, going out with friends […] is difficult. Also, for eating something out in good company, I don’t go, I stay at home”*) and lack of social acceptance and awareness of swallowing problems (*“By now people I […] see know about it and they wait to see the results, if I manage or not [to swallow] […] they are worried”*). Thirteen participants (52%; quotations n = 14, 26%) who did not feel limited in social occasions identified personal and social acceptance of OD as resources for positive adjustment:

“No, [the swallowing problem] did not cause troubles [in my interpersonal relationships], but when I eat with someone, I am very slow, anyway […] if they [other people] do not adjust to it, I can live with it.”

“My swallowing problem does not interfere […] I’m lucky for the relational system I have, I do not have moments of solitude that force me to concentrate on my condition.”

Table 3 provides an overview of participants’ perceived challenges in using healthcare services for OD diagnosis and management, and potential solutions (question 8). Twenty-two participants (88%) answered this question, with 38 extracted quotations. While around half of them could not identify any obstacle in accessing healthcare services, ten (40%) perceived services as inappropriate to meet their needs, due to the lack of patient-centred OD care networks that forced them to consult multiple specialists in different hospitals. Less frequently mentioned obstacles were personal coverage of healthcare expenses, to avoid the waiting lists characterizing public services; inappropriate supplies for PEG management; and difficulties in putting SLP suggestions into practice during daily life.

**Table 3:** Categorization of perceived obstacles and potential solutions in the use of healthcare services (question 8)

Category	Subcategory	n (%)	Ref (%)	Citations
Absence of obstacles	–	12 (48%)	13 (34%)	“Everything is perfectible […] but […] I would say it is a healthcare system which works […] I feel cared for”
Obstacles		10 (44%)	17 (45%)	
	Patient-centred services	6 (24%)	7 (18%)	“I went to examinations, no one told me anything—Everything is fine, everything is fine. In the meantime, I had the [swallowing] problem. […] If you saw liquids cannot be swallowed, there should be a problem and then [doctors] said—No, the throat is fine.”
	Swallowing network	5 (20%)	5 (13%)	“Finding in one hospital all the doctors who can take care of you […] it’s not possible to go to one hospital, then to another one, then in another, make an appointment and they don’t overlap, it becomes a mess”
	Timely assistance	3 (12%)	3 (8%)	“When they say, for instance, the cardiologist in eight months, I put my hands in my pocket, luckily I can afford it, I pay, and I go wherever I need to”
	Adequate devices	1 (4%)	1 (3%)	“They mistook a couple of times to send me the [syringe], the thread of the screw was not right, so the syringe could not be inserted, and I needed to drink [with the PEG]”
	Lack of guidelines	1 (4%)	1 (3%)	“We are doing what you tell us and what we can for the management. […] Every day we find ourselves adapting things, food, modalities, timing, and so on. […] It’s not a critique, but […] we don’t have […] a protocol, a guideline”
Solutions		5 (20%)	8 (21%)	
	Patient-centred services	3 (12%)	3 (8%)	“When you face a serious disease […], you need a cure but also you need to feel cared for a lot
	Swallowing team	2 (8%)	3 (8%)	“You should work on it [dysphagia and related complications] in team”
	Adequate devices	1 (4%)	2 (5%)	“A card [to] […] carry these things [PEG equipment] easily on a plane [and] be sure one hundred per cent that this stuff arrives where I go”

n = number of participants who cited the category/subcategory. Ref = number of text units retrieved for each category/subcategory

Some participants (n = 5, 20%) proposed solutions to overcome the described obstacles, such as patient-centred services, and higher attention to the person, besides their medical conditions. They also proposed to build dysphagia care teams, composed of all the professionals involved in OD management, and the availability of adequate devices for PEG management, especially during travels.

### Perceived Care Resources

Individual resources in dealing with OD-related challenges were explored by asking participants about their coping strategies and personal capabilities; social resources by inquiring about social and healthcare support. Among personal resources, coping strategies were described by twenty-two (88%) interviewees with 97 extracted quotations. Problem-focused coping was prominent (n = 22, 88%); it included improvement of meal management through changes in eating behaviour (e.g., eating smaller bites or at a slower pace), food choices (naturally easy-to-swallow food), daily routine of meal preparation and consumption, as well as eating patterns to participate in social gatherings. Almost half of the participants (n = 10, 40%) reported meaning-focused coping strategies, mostly referring to personal and social acceptance and positive adjustment to social occurrences. Only one participant (4%) reported using avoidance coping strategies to divert attention on goals related to life domains other than health. Table 4 shows the frequency and percentage distribution of answer units and participants across subcategories, with exemplary quotations.

**Table 4:** Categorization of coping strategies in dealing with OD (question 2)

Category	Subcategory	n (%)	Ref (%)	Citations
Problem-focused coping		22 (88%)	83 (86%)	
	Meal management	22 (88%)	68 (70%)	“Eating smaller bites, chewing them more and swallowing, and trying not to have anything else in my throat”
				“When I choose the bread, I prefer the soft one rather than the dry one, rather than crackers or breadsticks”
				“I need to prepare meals in advance […] when I arrive home, I heat them up”
	Social behaviours	9 (36%)	15 (15%)	“I leave the plate [when others have ended eating] […] I eat calmly but at a certain point when everybody has finished, I feel compelled to end as well”
Meaning-focused coping		10 (40%)	13 (13%)	
	Positive adjustment/acceptance	9 (36%)	12 (12%)	“I take my time, I consume [meals] in a different way, but I must not feel excluded from the activity, socially from going out with friends or from a dinner with many people”
	Social acceptance	1 (4%)	1 (1%)	“[The swallowing problem] has become normal among almost all [my acquaintances]”
Avoidance	–	1 (4%)	1 (1%)	“I try to think [about my swallowing problem] the least I can and to concentrate on other things, like my family, organizing a trip or going out”

n = number of participants who cited the category/subcategory. Ref = number of text units retrieved for each category/subcategory

Answering question 3, the majority of participants (n = 16, 64%) ascribed these solutions to their personal experience (“*I found the solutions by myself*”; quotations n = 9, 31%), while 8 (32%) received suggestions from health professionals *(“The SLP gave me these instructions”*; quotations n = 9, 31%), other persons with OD (n = 2, 8%; “*Another patient told me that [to use a chewing gum]*”; quotations n = 2, 7%) or family members (n = 2, 8%; “*My wife [found] the solution for it [the eating problem]*”; quotations n = 2, 7%).

Among personal resources, 21 (84%) participants (38 extracted quotations) identified personal capabilities (i.e., personal characteristics that shape the way individuals perceive life circumstances and act on them) which helped them cope with OD (Table 5). Ten interviewees (40%) cited resilient behaviours (e.g., adjusting to problems, transforming difficulties into opportunities) and hardiness, seven (28%) mentioned problem-focused coping, three (12%) quoted emotion-focused coping and the cultivation of hope and optimism, while only one (4%) reported avoiding the problem.

**Table 5:** Categorization of perceived personal capabilities (question 4)

Category	Subcategory	n (%)	Ref (%)	Citations
Resilient behaviours and hardiness	–	15 (60%)	21 (55%)	“Often […] what seemed a tragedy turned out to be a strength”
				“My steadfastness”
Problem-focused coping		7 (28%)	8 (21%)	
	Problem appraisal	4 (16%)	4 (11%)	“I try to rely on my reasoning […] to rationalise”
	Problem management	4 (16%)	4 (11%)	“You always must face the problem, you must solve it. I commit a lot”
Emotion-focused coping	–	3 (12%)	3 (8%)	“I try to be always calm”
Hope/optimism	–	3 (12%)	5 (13%)	“Maybe my optimism […] optimism is natural and luckily is my way of seeing things, and so I’m lucky”
Avoidance	–	1 (4%)	1 (4%)	“Erasing […] the problem because […] it gets me anxious […] I’m the kind of person who solves problems and cancels them if I can, otherwise I don’t think about them”

n = number of participants who cited the category/subcategory. Ref = number of text units retrieved for each category/subcategory

Social resources were reported by most participants (n = 23, 92%; 32 extracted quotations). They referred to the support received by informal caregivers and healthcare services. Most interviewees (n = 16, 64%) mentioned their family members or other informal caregivers as resources providing support. More specifically, eight participants (32%; quotations n = 8, 25%) referred to practical support: *“Yes, [my family] do the cooking, they help me finding solutions, trying […] other food to swallow. It’s a game: together we find something”*. Seven participants (28%; quotations n = 9, 28%) felt supported on the emotional side *“[My husband] sometimes tries to be funny—What are you doing?—[when I cough] […] he tries to… [downplay]”.* Five participants (20%; quotations n = 5, 16%) described the efforts of family members in adapting to their dietary needs *“They adjust to what I can eat”*. Support received from significant others, such as friends and colleagues, was more rarely mentioned (n = 2, 8%, quotations n = 2, 6%) *“Everyone tries to give me courage and to feel close as well […] my closest friends, those I, let’s say, confide in, they are really close to me”.* Five participants (20%, quotations n = 5, 16%) complained about the lack of family support *“There is no one who supports me […] II support others [family members]”.* Finally, three participants (12%; quotations n = 3, 9%) reported to be completely autonomous, thus not needing any support *“[Help is unnecessary] I’m autonomous enough”*.

Twenty-four (96%) interviewees reported on the support received by healthcare services (35 extracted quotations). Most participants (n = 22; 88%; quotations n = 33, 94%) expressed appreciation, specifically referring to the availability of swallowing specialists *“An answer was always there, and possibly in the shortest times”* (n = 5, 20%; quotations n = 6, 17%), the adequate offer of diagnosis *“I was referred here [Phoniatric clinic] […] by my oncologist”* (n = 10, 40%; quotations n = 11, 31%), rehabilitation pathways *“They helped me [understand] […] whether [the disorder] worsened, got better, or remained stable*” (n = 8, 32%; quotations n = 8, 23%), and the timely provision of practical instructions on feeding modalities and swallowing management *“I started understand which stuff were swallowed better than others”* (n = 6, 24%; quotations n = 8, 23%). Only two (8%) participants (quotations n = 2; 6%) did not feel supported by healthcare services in any respect concerning OD *“It’s a country that does not have a system […] you are alone”*.

Finally, twenty-four (96%) participants elaborated on the impact of OD on their view of life and social relations (25 quotations extracted). Around half of them (n = 13, 52%; quotations n = 13, 52%) did not perceive any relevant impact *“For me [everything] is the same as before”.* The other 11 interviewees (44%; quotations n = 12, 48%) instead identified positive and negative changes in their attitude towards life and others*.* Seven participants (28%; quotations n = 7, 28%) experienced a shift in life priorities and values:

“At the moment it’s difficult to see the future […], even if the cancer experience told me you must live the day, still a little […] planning […] I feel too much concentrated on the present and on what I’m passing that I fear thinking what I will do in six months, how I will do it, and whether I will manage to do it.”

Three (12%; quotations n = 3, 12%) attributed a greater importance to mealtime:

“It made me understand […] that […] also mealtime moment is something that should be taken cared of more, rather before it was about swallowing and tasting or not what was in your mouth, now in addition you should pay attention on how and what you swallow.”

Two focused on empathy from others *“There are people who understand, and it’s nice. Some people minimize, and this is not nice”* (8%; quotations n = 2, 8%)*.*

## Discussion

The results of this study highlighted that people living with chronic OD experience multifaceted daily challenges, that extend beyond the physical domain to cover practical, social, and psychological issues. Besides challenges, personal and social resources were emphasized by the participants. The typologies of perceived challenges are consistent with previous findings on the daily experience of individuals with OD [13]. The investigation of care resources instead represents a new perspective in OD literature, which may offer researchers and practitioners a more comprehensive and person-centred understanding of patients’ positive and negative life experiences. An adequate sample of Italian adults diagnosed with OD participated in the study, bringing insights into a social and cultural context rarely explored in the literature on dysphagia [13]. The modified framework approach to the analysis of qualitative data allowed us to connect the detailed descriptions of participants’ daily experiences with existing and well-established theoretical frameworks.

### OD-related Challenges

The daily challenges reported by people with chronic OD in the semi-structured interviews were categorized and linked to the Supportive Care Framework adapted to OD [13]. Participants reported physical, practical, social, psychological, and healthcare challenges, highlighting the need to look beyond physical concerns for comprehensive person-centred care. These findings are in accordance with expert opinions [45], quantitative evidence [46], literature reviews [13], and other qualitative studies involving persons with dysphagia [10]. In line with previous studies, participants acknowledged changes in their social roles. Nevertheless, around half of them did not report limitations in family and social life, suggesting that supportive relationships may help to adjust to chronic OD.

### Care Resources

The possibility of attaining positive adjustment to a chronic health condition is well described in the psychological literature [41]. It was however only sparsely investigated in relation to OD. Our findings suggest that people who deal with relatively stable OD may attain a satisfactory adaptation to their condition and experience meaningful lives, as witnessed in a recent autobiographical story published as personal opinion [47]. Consistently with the ICF framework [16], participants mentioned personal and social resources (or facilitators in the ICF language) which helped them deal with OD. Personal resources encompassed all coping strategies described in the theory of Stress and Coping [21], as well as personal capabilities representing well-established psychological constructs such as resilience [22], hardiness [23], and the cultivation of hope [26] and optimism [27]. Among social resources participants mentioned both family members and significant others, underscoring the importance of social connections in dealing with health problems [28]. Differently from a previous study conducted in Australia [11], participants did not mention patients’ associations as a source of social support, highlighting a relevant gap between healthcare services’ assistance and patients’ daily life context. While acknowledging the valuable support of healthcare services, participants claimed the implementation of patient-centred care models and the creation of structured “swallowing teams” including all relevant health professionals involved in dysphagia care [48].

Finally, from the perspective of the CSM [30, 31], the challenges identified by the participants mostly referred to the consequences of OD (*illness consequences*) in daily life. Through the integration of the experienced consequences with perceived OD severity (*illness identity*), chronic OD course (*illness duration*), and perception of personal control on OD (*illness controllability*), participants adopted coping strategies and mobilized personal and social resources to adapt to the situation. Acknowledging that the cross-sectional design of this study prevents causality inferences, our results suggest the importance of promoting adaptive coping strategies based on the person’s perception of OD severity to support resource building.

### Strengths, Limitations, and Future Directions

This study represents a novel investigation of the lived experience of people with chronic OD. The categorization of qualitative data based on existing frameworks allowed for connecting participants’ subjective experiences with well-established theories and constructs. Results have also practical implications, as they can offer clinicians and researchers useful information to better understand patients’ perspectives and incorporate positive adjustment promotion into OD care.

The present study is not exempt from limitations. The diagnosis of OD shared by all participants derived from a heterogeneous range of diseases. We acknowledge that different pathophysiological mechanisms and disease stages may lead to different OD severity, with diverse implications on daily activities and social participation. Moreover, in the specific context of neurodegenerative diseases, the comorbidity with motor and cognitive impairments may further negatively influence daily experience, by reducing autonomy and enhancing the need for assistance. Although a quantitative analysis of the differences in reported themes based on diagnosis, OD severity, and OD onset was outside the scope of the present study, a joint examination of both the extracted quotations and notes and minutes collected throughout the study allowed for some additional considerations: (i) participants with neurodegenerative diseases tended to more often report as challenges themes related to autonomy and voice symptoms; (ii) participants with head and neck cancers tended to present more severe OD and to report awareness of the negative implications of their swallowing symptoms on their daily mealtimes, while being well-aware of their personal and social resources. Studies focused on single clinical populations are however needed, to more deeply delve into disease-specific sets of perceived challenges and resources.

As concerns sociocultural and environmental issues, all the participants lived in Northern Italy regions characterized by similar healthcare systems; different comments and suggestions could derive from interviews with participants living in other Italian regions or different countries, due to differences in healthcare organization and access, family structure and culture. Moreover, due to the COVID-19 pandemic and based on participants’ preferences, interviews were conducted in different environments (outpatient clinic or participants’ home) and through different communication modes (either in-person or through video-call). Although the appropriateness of the interview setting was checked to grant comfort and privacy, the potential influence of the location on the study results is unknown.

The cross-sectional design of the study prevents any causal and time-related interpretation of the findings. Future longitudinal studies are needed, to broaden knowledge on the mechanisms underlying positive adjustment to chronic OD in the context of different pathologies and across countries Finally, while the working analytical framework developed from the interviews was thoroughly discussed and refined based on the clinical and research perspectives of the research team members, pragmatic constraints prevented us from performing member checking with participants.

## Conclusions

Results from this study suggest the importance of broadening the healthcare models and strategies beyond the physical domain; evidence collected among participants living with chronic OD can be potentially extended to people dealing with other kinds of clinical conditions. Besides daily challenges, a broad set of personal and social care resources were identified, highlighting their importance and role in designing personalized care and rehabilitation strategies.

## Data Availability

Data is available upon request to the corresponding author.

## Appendix 1

### Semi-structured Interview Guide

As this is a conversational-style interview, not all questions may be asked.

1. What are your main problems, limitations, and needs in daily managing your swallowing problem?
2. How do you cope with these problems and limitations? Which solutions have you found?
3. Have you found these solutions on your own or were they suggested by someone? By whom?
4. What are the most important personal capabilities that you use to cope with your swallowing problem?
5. How does the swallowing problem limit your family life? And your social life?
6. Do family members/Does the person who assists you help to cope with your swallowing problem? How?
7. Do healthcare services help you cope with your swallowing problem? How?
8. What are the main obstacles you experienced using healthcare services? What would you suggest for improving them?
9. Have you changed the way you see life and other people because of your swallowing problem? If yes, how?

## Appendix 2

Proportion agreement and instances of disagreements between coders for each interview question.

**Table:**

Question	N extracted text-units	N conjunctively extracted text-units	Proportion agreement	Disagreements (N; %)
Question 1	105	90	86%	15; 14%
Question 2	97	81	84%	16; 16%
Question 3	29	28	97%	1; 3%
Question 4	38	35	92%	3; 8%
Question 5	53	47	89%	6; 11%
Question 6	32	30	94%	2; 6%
Question 7	35	34	97%	1; 3%
Question 8	38	34	89%	4; 11%
Question 9	25	25	100%	–
